# Clinical Significance of Atypical Squamous and Glandular Cell Lesions in Cervical Smear Screening Cytology: A Two-Year Follow-Up Study in an Iranian Population 

**DOI:** 10.30699/IJP.2024.2020412.3242

**Published:** 2024-10-29

**Authors:** Narges Izadimood, Fatemeh Nili, Soheila Sarmadi, Houra Omdeh Ghiasi, Elham Mirzaeian

**Affiliations:** 1 *Department of Pathology, Yas Hospital Complex, Tehran University of Medical Sciences, Tehran, Iran*; 2 *Department of Pathology, Imam Khomeini Hospital Complex, Cancer Institute, Tehran University of Medical Sciences*; 3 *Tehran University of Medical Sciences, Tehran, Iran*; 4 *Department of Pathology, Shariati Hospital, Tehran University of Medical Sciences, Tehran, Iran*

**Keywords:** Cervix, Cytology, Atypical squamous cell, Atypical glandular cell, Follow-up Study

## Abstract

**Background & Objective:**

Atypical squamous cells (ASC) are the most common epithelial abnormalities found in cervical cytology reports. The clinical significance of ASC and atypical glandular cells (AGC) varies, making clinical management and follow-up challenges.

**Methods:**

All women diagnosed with ASC or AGC in the past 4 years and referred to a tertiary hospital were included. The study evaluated regression, persistence, or progression to significant abnormalities over a two-year follow-up period.

**Results:**

Out of 22,386 cervical cytology smears, 208 (4.8%) patients were diagnosed with ASC (ASC-US: 3%, ASC-H: 1.8%) or AGC (0.25%). Among ASC-US patients with documented follow-up, 11 (46%) showed significant abnormalities, while 13 (54%) showed insignificant abnormalities. In the ASC-H group, with available follow-up, 20 (72%) showed significant abnormalities, and 8 (28%) showed insignificant abnormalities. When considering ASC-US and cervical intraepithelial neoplasia 1 (CIN 1) as low-grade lesions, 19 (31%) patients with ASC-H had low-grade, and 13 (69%) had high-grade abnormalities. In the ASC-US group, 10 (99%) patients had low-grade lesions, while only 1 (1%) had high-grade lesions. Among AGC, not otherwise specified (NOS) patients with follow-up, 17 (65%) had significant lesions, and 9 (35%) had insignificant lesions. All 13 patients with AGC, favor neoplastic (FN)/adenocarcinoma in situ (AIS), showed significant lesions.

**Conclusion:**

While patients diagnosed with ASC-H and AGC are at a higher risk for significant lesions, ASC-US patients may also develop significant lesions. Thus, ASC-US is clinically significant, and these patients should be closely monitored.

## Introduction

Cervical cancer is the fourth most common malignancy in women. In 2020, about 90% of new cases and deaths were reported from low and middle-income countries (1-4). Incidence and mortality rates in countries with low human development index (HDI) are three and six times higher than in the countries with high HDI, respectively (5).

Cytologic evaluation of cervicovaginal smears for screening of precancerous lesions was introduced in 1928 by George Papanicolaou. Over the decades, the defining terminologies have been evolved (2). Despite its limitations, programmed cervical cytology screening in developed countries has been successful in reducing the prevalence, morbidity, and mortality of cervical cancer (6). Given the significant pathogenic role of HPV in development of cervical intraepithelial lesions and invasive carcinomas (7), currently, HPV testing has been highlighted as the first screening method. However, due to a higher cost compared with cytologic smears, development of this method has limitations in low-income countries. Meanwhile, cytologic diagnosis is the next step of management and follow-up approach in patients with a positive HPV test for genotypes other than 16 and 18 (8-10). 

Based on the last edition of the Bethesda system, epithelial cell abnormalities are divided into two categories: squamous or glandular cells. In each group, the subcategory of ASC (atypical squamous) and AGC (atypical glandular cells) defines those cases in which cellular abnormalities are more significant than reactive or inflammatory changes but do not completely fit the criteria of squamous intra-epithelial lesion or glandular neoplasia (11).

ASC is further subdivided into ASC-US for reporting the lesions that are suggestive of squamous intraepithelial lesions but lack definite criteria and ASC-H for those that are suspicious of HSIL but don't have the full criteria for it (11).

ASC is the most common epithelial abnormality in cytologic reports. The clinical significance of these lesions is also variable, so the clinical management and follow-up could be challenging. The aim of this study is to investigate the prevalence and clinical significance of these diagnostic categories along with 2-year follow-up of the patients in a referral center in an Iranian population.

## Material and Methods

In this retrospective study, all the women previously diagnosed with ASC or AGC during the last 4 years referred to Yas Hospital complex were included. The cytologic slides were reviewed by two expert gynecopathologists. On the two-year follow-up of the patients, repeated abnormal cytology or biopsy-proven squamous or glandular abnormalities, including low and high-grade SIL (CIN 1-3), adenocarcinoma in situ, squamous cell carcinoma, invasive adenocarcinoma, and endometrial hyperplasia/ carcinoma were considered significant abnormalities. Any other inflammatory, reactive, or metaplastic lesions were considered insignificant lesions. In other analyses, ASC-US and CIN 1 were considered as low-grade, HSIL (CIN 2, 3), SCC, AIS, and adenocarcinoma as high-grade lesions. The prevalence of progression to significant or regression to insignificant lesions, as well as low-grade and high-grade abnormalities, were evaluated and compared in ASC-US, ASC-H, AGC Not otherwise specified (NOS), and AGC favor neoplastic (FN)/ AIS subcategories.

For data analysis, SPSS software version 23 was used. P-value > 0.05 was considered significant.

The study was conducted retrospectively, without any new interventions or costs to the patients. Only the archived documents and slides were used for data analysis. The patients confidentiality was maintained.

## Results

A total of 23886 patients were referred to our center for cervical cytologic examination over a four-year period. Among these, 208 cases were diagnosed as "ASC or AGC” and included in this Study. The patients’ mean age was 42.8 (range: 18-84).

After reviewing the slides, the prevalence of ASC and AGC subcategories was found to be 4.8% (ASC-US: 3%, ASC-H: 1.8%) and 0.25%, respectively (Table 1). 

**Table 1:** Frequency and mean age of the patients in ASC (Atypical squamous cell) and AGC (Atypical glandular cell) subcategories in the whole population. ASC-US (Atypical squamous cell of undetermined significance), ASC-H (Atypical squamous cell, cannot exclude HSIL), AGC, NOS (Atypical glandular cell, Not otherwise specified), AGC, FN (Atypical glandular cell, favor neoplastic)/ Adenocarcinoma in situ (AIS)

Final Diagnosis	Number	Frequency	Mean age
ASC-US	74	3%	36.5
ASC-H	44	1.8%	44
ASC-H+AGC	1	0.0004%	
AGC, NOS	20	0.08%	48.7
AGC, Endocervical	20	0.08%	43
AGC, endometrial	7	0.02%	53.1
AGC, FN/ AIS	15	0.06%	50

A total number of 147 included cases were less than 50, and the remaining 56 cases were more than 50 years old. ASC-US was the most common diagnosis in the patients younger than 50, and AGC was the most common abnormality in cases older than 50 years (*P*<0.001).

The patients were managed based on ASCCP guidlines (American Society of Cervical Cancer Prevention 2012). The First follow-up result (0-12 months after the initial diagnosis) was documented for 103 patients.

**Fig. 1:**
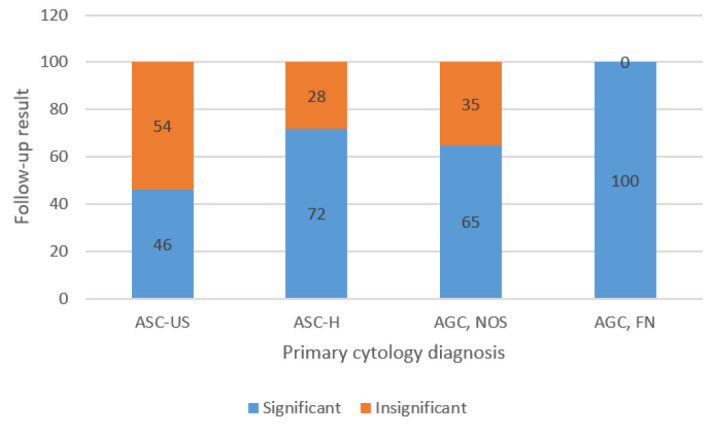
Frequency of significant and insignificant lesions on the first follow-up result of the different ASC and AGC categories

A follow-up report was available for 24 cases in the patients with ASC-US reports. Eleven (46%) of them showed significant and 13 (54%) insignificant abnormalities. In the ASC-H group, follow-up was available for 28 patients. Twenty (72%) patients revealed significant and 8 (28%) insignificant abnormalities (Figure 1). The frequency of different diagnoses in these subcategories is shown in Table 2.

**Fig. 2:**
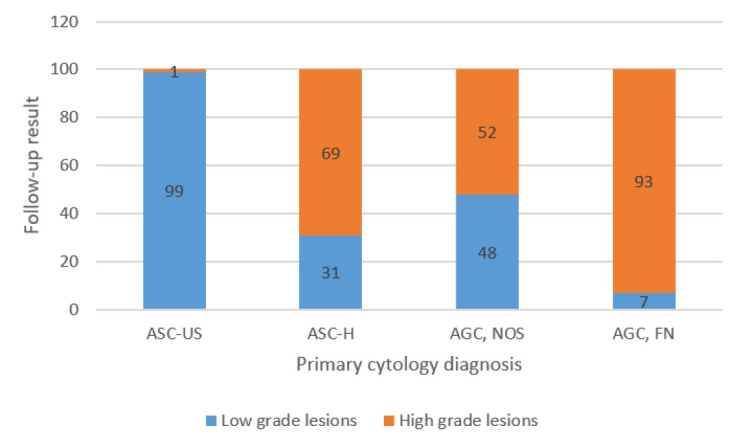
Frequency of the low and high-grade lesions on the first follow-up result of the different ASC and AGC categories

Considering ASC-H, HSIL, CIN2, CIN3, and carcinoma as High grade and LSIL, CIN1, and ASC-US as low-grade lesions, re-analysis of the patients showed 19 (31%) patients with ASC-H diagnosis with low grade and 13 (69%) high-grade abnormalities. In the ASC-US group, 10 (99%) of patients showed low grades, and only one (1%) revealed high-grade lesions. The difference between ASC-US and ASC-H cases was statistically significant (*P*=0.002) (Figure 2).

**Table 2:** Frequency of different diagnoses on the first follow-up in the ASC-US, ASC-H, and ASC-H+ AGC. FN groups

	Primary Cytology Diagnosis		
	ASC-US	ASC-H	ASC-H + AGC.FN/ AIS
Follow-up result No follow up	50 (67%)	16 (36%)	0 (0%)
Insignificant	13(16.7 %)	8 (17.2 %)	0 (0 %)
ASC-US	2 (2.6 %)	0 (0%)	0 (0 %)
ASC-H	0 (0 %)	2 (4.5 %)	0 (0 %)
LSIL	0 (0 %)	1 (2.2 %)	0 (0 %)
HSIL	0 (0 %)	1 (2.2 %)	0 (0 %)
CIN1	2 (2.7 %)	2 (4.5 %)	0 (0 %)
CIN 1,2	0 (0 %)	2 (4.5 %)	0 (0 %)
CIN 2,3	0 (0 %)	1 (2.2 %)	0 (0 %)
CIN 3	1 (1.3 %)	3 (6.8 %)	0 (0 %)
Carcinoma	0 (0 %)	6 (13.6 %)	0 (0 %)
Others	6 (7.3 %)	2 (4.6 %)	1 (100 %)
Total	74 (100 %)	44 (100 %)	1)100%)

Among the patients with AGC, follow-up for 26 cases was available. Seventeen patients (65%) showed significantly, and 9 (35%) revealed insignificant lesions (Figure 1). Available follow-up for 13 patients with AGC, FN/ AIS revealed significant lesions in all of them (*P*=0.018). Table 3 shows frequency of the different diagnoses in AGC subcategories.

**Table 3:** Frequency of different diagnoses in AGC subgroups (NOS and FN) on the first follow-up.

Primary cytology diagnosis		
AGC.FN/ AIS	AGC.NOS	
**2 (13 %)**	21 (44 %)	**Follow-up result** **No follow up**
**0 (0 %)**	9 (18.3 %)	**Insignificant**
**0 (0 %)**	1 (2.1 %)	**ASC-US**
**0 (0 %)**	0 (0 %)	**ASC-H**
**1 (6.6 %)**	1 (2.1 %)	**AGC**
**0 (0 %)**	2 (4.2 %)	**CIN 1**
**0 (0 %)**	1 (2.1 %)	**CIN 2,3**
**1 (6.6 %)**	1 (2.1 %)	**CIN3**
**1 (6.6 %)**	3 (6.3 %)	**AIS**
**0 (0 %)**	1 (2.1 %)	**VAIN3/ CIN3**
**0 (0 %)**	1 (2.1 %)	**Endometrial hyperplasia**
**8 (53 %)**	3 (6.3 %)	**Carcinoma**
**2 (13 %)**	3(6.3%)	**Other**
**15 (100 %)**	47 (100 %)	**Total**

Among the patients with significant lesions in AGC, NOS group, 8 (47%) revealed low-grade, and 9 (53%) revealed high-grade lesions. The frequency was 92% high-grade versus 8% low-grade lesions (*P*= 0.042) (Figure 2).

The second follow-up (12-36 months) was available for 30 patients. Twelve patients revealed insignificant lesions, and 18 had significant lesions. Only 2 patients with insignificant lesions on the first follow-up showed significant lesions on the second follow-up.

On follow-up, 32 (44.4%) patients under 50 showed insignificant and 40 (55.6%) significant abnormalities. On the other hand, 23 (74.2%) of patients, more than 50, had significant lesions, and 8 (25.8%) revealed insignificant lesions. Although the older patients more commonly had a significant lesion on follow-up, the statistical difference was not significant (*P*=0.08)

The HPV test was performed on only two patients with ASC-US diagnosis. Both were positive for HPV 16 and had CIN1 and squamous cell abnormality of unknown significance on follow-up biopsy.

## Discussion

In this study, we reviewed the medical files of 208 patients with an initial diagnosis of ASC or AGC. Among the abnormalities, ASC-US showed the highest prevalence (3%), followed by ASC-H (1.8%), AGC. NOS (0.18%) and AGC, FN/AIS (0.06%). Previous studies on the prevalence of Pap smear abnormalities have reached similar conclusions. In the vast majority of these studies, ASC was the most common interpretation in epithelial abnormality and accounted for about 4% of cervicovaginal cytological diagnoses worldwide (12, 13). 

Our results indicated that patients older than 50 years of age were more likely than patients younger than 50 years of age to have significant lesions in follow-ups, although this difference was not statistically significant. In a study on ASC-US patients, there was no significant difference in the occurrence of significant lesions during follow-up, as patients were divided into two groups under 35 and older (14). Some studies have shown that the prevalence of high-grade lesions in postmenopausal women is lower than in premenopausal women (15). The reason is the presence of artifactual changes in postmenopausal women such as drying artifact, inflammation, reactive metaplasia, the presence of many bare nuclei, pseudo parakeratosis, etc., all of which induce over-diagnosis of atypical squamous cells (12, 16). In a study of patients with an initial diagnosis of AGC, it was found that although clinically significant lesions are more common in those under 60 years of age, high-grade uterine lesions are more common in those over 60 years of age (17). As can be seen, the results of our study on the effect of age on patient follow-up results are inconsistent with those of other studies. This finding may be due to two reasons: First, the mean age of people with higher-grade lesions such as ASC-H or AGC, FN/AIS in this study was higher than low-grade lesions. The second reason may be our center's sufficient mastery in differentiating pseudo-neoplastic and reactive lesions from real neoplastic lesions.

In our study, the results of the first and second follow-ups were made within 12 and 36 months after the diagnosis, respectively. The majority of significant lesions were detected during the first follow-up, and the results of the second follow-up had little effect on the detection of clinically significant lesions. The results of the third follow-up, available only for 3 patients, did not provide any additional information. 

In a study, the average time required for high-grade lesions to appear was reported to be about 38 months, and it was stated that no results changed after 7 years of follow-up (13). Thus, the authors concluded that follow-ups longer than 7 years will not change anything, and a maximum follow-up of 7 years is sufficient (13). Another study showed that in lesions with initial diagnosis of AGC, the majority of clinically significant lesions were diagnosed within 1 year, and in other cases, the median time between diagnosis of AGC and finding a clinically significant lesion was 37 months (18). In another study of patients with an initial diagnosis of ASC-US, the mean time to detect lesions such as LSIL or HSIL was 12.1 months, and the mean time to achieve a normal diagnosis (one colposcopy result and two consecutive normal Pap smears) was approximately 13.9 months (19). 

Another point was about the optimum time to discover significant results in follow-ups. Some previous studies of follow-up results of patients with abnormal Pap smears found that when a second smear is prepared immediately, or a short time from the first smear, it does not help much in disease management (19, 20). It is suggested to get a second smear 6 months after the first smear (21). On the other hand, Emerson et al. indicated that if a biopsy was performed at follow-up earlier than 6 months after the Pap smear, the likelihood of LSIL reports was higher. Therefore, the best time for the first follow-up after the diagnosis of an abnormal smear is 6 months (13). In our study, the majority of first follow-ups were performed 6 months after the abnormal Pap smear was diagnosed. As mentioned, in 2 patients who had insignificant lesions in the first follow-up, the second follow-up (in one patient 9 months later and in the other 12 months later) resulted in the discovery of two new significant lesions. Therefore, these findings may support the hypothesis of previous studies that if follow-ups are performed later (for example, after 6 months), it may help us to detect significant lesions. In addition, this can prevent over-diagnosis of repair changes after a Pap smear test and misunderstanding of them as pre-malignant lesions.

In this study, of the patients with ASC-US whose follow-up results were available, 54.2% showed clinically insignificant changes, and 45.8% showed significant lesions. Previous studies have shown that more than half of patients with an initial diagnosis of ASC-US will not show significant lesions at follow-up, meaning they are more likely to return to normal (19, 20, 22). In 2002, Emerson *et al.* followed 643 patients diagnosed with ASC-US for 9 years. They stated that 43.9% of these individuals eventually showed clinically significant changes, most of which were mild dysplasia (SIL) (13). In their study, the risk of spontaneous regression in ASC-US was reported 68% (13). In another study by Negri et al., the prevalence of significant lesions in patients diagnosed with ASC-US was about 30%, and it was also declared that women with ASC-US were 5 to 10 times more likely to have neoplastic lesions than women with normal Pap smears (23). In two other similar studies, the prevalence of these lesions in ASC-US was reported to be around 50% and 51% (16, 24). In our study, 15.2% of patients with an initial diagnosis of ASC-US reveal unremarkable follow-up biopsy indicative of spontaneous regression. Lack of access to follow-up results of ASC-US patients may be the potential reason why the prevalence of spontaneous regression rate in our study is lower than others. It seems that patients with other initial abnormalities rather than ASC-US are more likely to perform follow-ups due to the greater sensitivity of both patients and physicians. 

Various studies have been performed on the follow-up of individuals with an initial diagnosis of ASC-H and its comparison with the ASC-US group. Chieng et al. reported the prevalence of normal and abnormal lesions in ASC-H to be 32.1% and 68.9%, respectively (17). In a study in Iraq of women diagnosed with ASC, the prevalence of LSIL and HSIL was reported significantly higher in the ASC-H group than in the ASC-US group (22). In another study of a number of patients diagnosed with ASC-H, Selvaggi et al reported 25% of normal follow-up outcomes and 75% of abnormal outcomes (15). In the present study, the rate of significant lesions in the ASC-H group was 71%, which was in concordance with previous studies and was higher than the ASC-US group (45.8%), but this difference was not statistically significant. However, due to the fact that the p-value level is close to 5%, it seems that could be due to the low sample size of this study.

The follow-up of AGC, NOS, and AGC, FN/ AIS patients showed 65.4% and 100% significant lesions, respectively. This difference between the two groups was statistically significant. This is similar to the results of previous studies, which indicated that the prevalence of clinically significant lesions was significantly higher in patients diagnosed with AGC.FN than in patients with AGC, NOS (25). Swangsang *et al.* conducted a study on 63 patients diagnosed with AGC in 2008 (26). Follow-up results of these patients revealed 77.8% without specific abnormalities and 22% with significant abnormal lesions. As noted, the risk of significant lesions has been highly variable in various studies. In our study, this risk is higher than in many studies in the world, which may be because studies on the history and development of atypical glandular lesions are much less and more difficult than ASC lesions. In addition, the prevalence of clinically significant lesions in the AGC depends not only on the study population and the overall risk of progression to cancer in that community but also on the age group of the study population (17). Another reason for the high number of significant lesions in the AGC group in our study compared to other studies may be the referral of this center and the large number of consultation cases with the diagnosis of AGC, NOS, and AGC, FN/ AIS.

A comparison of the two groups of AGC and ASC in the present study showed that the risk of significant lesions in the AGC group (76.9%) is higher than these lesions in the ASC group (59.6%). However, this difference between the two groups was not statistically significant. This confirms the results of other studies indicating that the ASC group is less valuable among the abnormal diagnoses presented in Pap smears despite being more valuable than normal cases. Many studies have finally concluded that ASC (even ASC-US) should remain a Pap smear diagnosis and should not be omitted, as it helps identify people at high risk for cervical dysplasia (13). Some authors believe in the overuse of the ASC-US term, suggesting that many people who are diagnosed with ASC-US may be part of the normal or SIL group with reactive changes (13). A study on the need for ASC-US to remain among Pap smear diagnoses found that the risk of a 2-year progression from ASC-US to HSIL was higher than normal but less than the progression of LSIL to HSIL. Therefore, ASC-US should be a separate category of Pap smear diagnoses (13). Other studies found that the likelihood of a significant lesion increases with the number of ASC-US diagnoses (19, 27). Most studies in this area believe that both the ASC-US and ASC-H groups identify patients at higher risk for progression to high-grade squamous intraepithelial lesions (HSIL) (22). Therefore, they should be maintained as two separate diagnoses among the diagnoses for Pap smear and patients with this diagnosis should be well followed and managed.

Various studies have emphasized the need for HPV testing, especially in cases of intermediate diagnoses such as ASC and especially ASC-US (12, 28). Many studies have emphasized that the follow-up of patients with HPV testing maybe even better in some cases than colposcopy if it is available (29). The last consensus on the management of patients with cervical intraepithelial abnormalities (2019 ASCCP) recommended a personalized risk-based management, considering the age, the current cytology and HPV results, and the past history of the patient. Based on the patient’s risk assessment of having or developing CIN3+, which is less or greater than 4%, recommendations for routine screening, 1 or 3-year surveillance, colposcopy, or treatment are considered (30, 31). Due to the high cost of HPV testing, most of the patients in our study were followed by colposcopy rather than an HPV test. Unfortunately, HPV test results were available for only 2 of the patients we studied, both with an initial diagnosis of ASC-US, and for both, HPV 16 was positive. Eventually, one developed CIN1, and the other had a squamous abnormality of uncertain significance. The lack of routine follow-up of patients with HPV tests and also the high cost of this test may be the biggest disadvantage of the management approach and follow-up of abnormalities of Pap smear tests in many countries, including Iran (32).

## Conclusion

Pap smear is still a precious screening test for cervical cancer. Although patients diagnosed with ASC-H and AGC are at a higher risk for significant lesions, ASC-US patients may also develop significant lesions. Thus, ASC-US is clinically significant, and these patients should be closely monitored, based on the best evidence available, it is better to follow up with the patients approximately 6 months after the first abnormal Pap smear result. There is still a need for further evidence-based recommendations with well-designed studies in the future.
